# Accelerating voxelwise annotation of cross-sectional imaging through AI collaborative labeling with quality assurance and bias mitigation

**DOI:** 10.3389/fradi.2023.1202412

**Published:** 2023-07-11

**Authors:** David Dreizin, Lei Zhang, Nathan Sarkar, Uttam K. Bodanapally, Guang Li, Jiazhen Hu, Haomin Chen, Mustafa Khedr, Udit Khetan, Peter Campbell, Mathias Unberath

**Affiliations:** ^1^ Department of Diagnostic Radiology and Nuclear Medicine, School of Medicine, University of Maryland, Baltimore, MD, United States; ^2^ Johns Hopkins University, Baltimore, MD, United States

**Keywords:** AI assisted annotation, AI-collaborative labeling, artificial intelligence—AI, CT volumetry, computed tomography, trauma, quantitative visualization, human in the loop (HITL)

## Abstract

**Background:**

precision-medicine quantitative tools for cross-sectional imaging require painstaking labeling of targets that vary considerably in volume, prohibiting scaling of data annotation efforts and supervised training to large datasets for robust and generalizable clinical performance. A straight-forward time-saving strategy involves manual editing of AI-generated labels, which we call AI-collaborative labeling (AICL). Factors affecting the efficacy and utility of such an approach are unknown. Reduction in time effort is not well documented. Further, edited AI labels may be prone to automation bias.

**Purpose:**

In this pilot, using a cohort of CTs with intracavitary hemorrhage, we evaluate both time savings and AICL label quality and propose criteria that must be met for using AICL annotations as a high-throughput, high-quality ground truth.

**Methods:**

57 CT scans of patients with traumatic intracavitary hemorrhage were included. No participant recruited for this study had previously interpreted the scans. nnU-net models trained on small existing datasets for each feature (hemothorax/hemoperitoneum/pelvic hematoma; *n* = 77–253) were used in inference. Two common scenarios served as baseline comparison- *de novo* expert manual labeling, and expert edits of trained staff labels. Parameters included time effort and image quality graded by a blinded independent expert using a 9-point scale. The observer also attempted to discriminate AICL and expert labels in a random subset (*n* = 18). Data were compared with ANOVA and post-hoc paired signed rank tests with Bonferroni correction.

**Results:**

AICL reduced time effort 2.8-fold compared to staff label editing, and 8.7-fold compared to expert labeling (corrected *p* < 0.0006). Mean Likert grades for AICL (8.4, SD:0.6) were significantly higher than for expert labels (7.8, SD:0.9) and edited staff labels (7.7, SD:0.8) (corrected *p* < 0.0006). The independent observer failed to correctly discriminate AI and human labels.

**Conclusion:**

For our use case and annotators, AICL facilitates rapid large-scale curation of high-quality ground truth. The proposed quality control regime can be employed by other investigators prior to embarking on AICL for segmentation tasks in large datasets.

## Introduction

Scalable artificial intelligence solutions require large datasets with high quality annotation, and for quantitative visualization tools, this has traditionally entailed prohibitively painstaking manual voxelwise labeling (1, 2). To date, few FDA-approved commercially available tools perform segmentation and quantification tasks (3–5). In 2018, The NIH, RSNA, ACR, and the Academy for Radiology and Biomedical Imaging Research held a workshop that stressed data scarcity as a major obstacle requiring approaches for high-throughput data curation (6) and spurred research efforts aimed at accelerating annotation in the domains of federated learning, active learning, artificial intelligence-assisted annotation (AIAA) with limited clicks and scribbles, semi-supervised learning, and synthetic data augmentation (7–18).

A simple AI collaborative labeling (AICL) method distinct from these approaches, involves training on a subset of manually labeled seed data, and then editing inference labels on unseen studies. Expert editing of automated labels was performed as a final annotation step for the public COVID 19–20 Lung CT Lesion Segmentation Challenge (19) dataset. Substantial time savings may be possible, especially for studies with extensive infiltrates, as studies with larger target volumes take longer to label (13). Another application of this collaborative approach involves editing inference labels and iteratively retraining a model in batches (20, 21). Time savings result with each batch as the model becomes more generalizable and there are fewer errors to correct. With these approaches, automation and complacency bias become potential concerns (22)- for example, labelers may be lulled into assuming automated labels are correct or second guess their own correct judgement.

**Figure 1:**
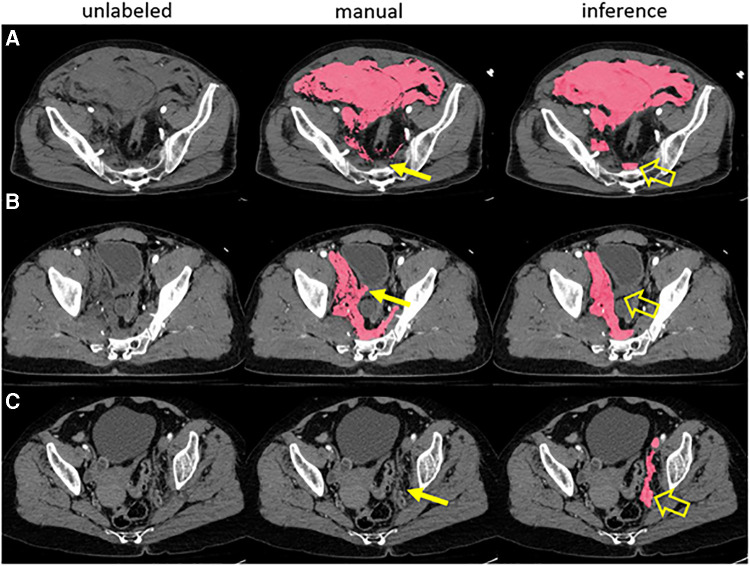
A prior pelvic hematoma segmentation algorithm correctly labeled hemorrhage in three examples with human error in manual training data (**A-C**). The expert missed a small volume of presacral blood in case A, inadvertently labeled a small segment of bladder wall in case B and missed a small volume of left pelvic sidewall hematoma in case C (thin arrows). The model, trained in 5-fold cross-validation, labeled these areas correctly in inference (open arrows).

A broader issue is the general fallibility of expert labelers (23) and the near-universal assumption in the published literature that manual ground truth is the ideal benchmark for determining algorithm performance (24). Taking COVID infiltrates as an example, annotators are faced with multifocality; highly variable shapes, locations, and volumes of infiltrates; widely differing attenuation values; irregular contours; and adjacent opacities such as atelectasis that can be mistaken for infiltrate. Even when using inference labels as a starting point, a mean Dice Similarity Coefficient (DSC) of only 0.70 was achieved between observers in the aforementioned COVID challenge (19). In a study of traumatic hemoperitoneum, a similarly complex task, mean test-retest DSC for manual annotator was also 0.70 (25). Algorithms will tend to ignore outliers in new data, and this is typically acknowledged as a flaw with respect to robustness. But when considering outliers related to mislabeling by humans, this vice of overfitting can become a virtue, as human inconsistencies may be ignored to arrive at a more uniformly satisfactory label (Figure 1). Thus, there is a possibility that an edited inference label with less-than-perfect overlap to a manual label may be qualitatively more suitable for use as ground truth.

As a “sanity check”, a qualitative or semi-quantitative label quality assessment could be performed by a blinded expert observer to determine whether manual or automated labels are the better ground truth for a given study. Semi-quantitative scores that are higher for edited automated labels empirically exclude detrimental automation bias. We are not aware of prior work in medical imaging segmentation that has employed this approach.

A recent scoping review of AI CAD tools for trauma found that while very large datasets have been developed for 2D detection tasks such as for pulmonary abnormalities and extremity factures on plain radiography, dataset sizes for torso hemorrhage-related pathology ranged between fewer than 100 to 778 patients (4, 25–31). Despite the perceived unmet need for hemorrhage-related quantitative visualization tools (32), due to challenges unique to the torso- namely small target to total volume ratios and complex pathology- deep learning (DL) algorithms yielding meaningful visual results for the chest, abdomen, and pelvis, have been late-comers in medical image analysis (33). A limited number of studies using small datasets have demonstrated an association between intracavitary torso hemorrhage volumes and clinical outcomes (25, 29, 34–36), with comparable or improved prediction compared to existing categorical grading systems. In 2021, Isensee et al., introduced nnU-net (37), an out-of-the-box self-configuring data-driven method which incorporates a 3D coarse-to-fine multiscale approach that has shown state-of-the-art performance on numerous public medical imaging datasets over bespoke multiscale methods.

A rapid-throughput labeling strategy using an AICL approach could make high-quality voxelwise annotation feasible for very large corpuses of CT data. Using 57 unseen trauma torso CT exams, we conducted this pilot study to determine time savings and annotation quality associated with AICL using nnU-net models trained with existing labeled seed data for several hemorrhage-related features- namely hemothorax, hemoperitoneum, and extraperitoneal pelvic hematoma. Two commonly employed labeling approaches- manual expert labeling, and manual labeling by trained staff with expert editing- are used for comparison. Label quality is compared using blinded assessment with a proposed 9-point RAND/UCLA Likert grading system.

## Methods

### Deep learning method

We trained nnU-net (37) in five-fold cross-validation using three existing datasets with voxelwise labeling of the feature of interest from previous pilot studies- a hemoperitoneum dataset of 130 patients, a pelvic hematoma dataset of 253 patients, and a hemothorax dataset of 77 patients. Cascaded (3D low-resolution to high-resolution) nnU-net yielded improved DSCs over prior bespoke methods for all three use cases (25, 29, 34, 38), with mean DSC increasing to 0.75 from 0.61 for hemothorax; to 0.67 from 0.61 for hemoperitoneum, and to 0.75 from 0.71 for pelvic hematoma.

### CT imaging

Trauma whole-body CT studies are performed at our institution on one of two trauma bay-adjacent scanners- a 64-section scanner (Brilliance; Philips Healthcare, Andover Mass- scanner 1), and a dual-source 128-section scanner (Siemens Force; Siemens, Erlangen, Germany- scanner 2). Studies are performed with 100 ml of intravenous contrast material [Omnipaque (iohexol; 350 mg/ml iodine)] at an injection rate of 5 ml/sec (scanner 1) or 6 ml/sec (scanner 2) for the first 60 ml followed by 4 ml/sec for the remainder. For penetrating trauma, we routinely administer rectal contrast (50 ml iohexol with 300 mg of iodine diluted in 1l of water) if abdominal penetration is suspected based on the location of entry and/or exit wounds. Arterial phase images are acquired from the thoracic inlet through the greater trochanters, followed by portal venous phase images through the abdomen and pelvis beginning just above the dome of the diaphragm. Images are archived at 3 mm section thickness.

### Dataset

Our primary aim was to determine time savings using AICL on new patients. For time analyses using comparison of means, we expected large effect sizes. Cohen recommended a D value of 0.2, 0.5, and 0.8 for low, medium, and high effect sizes respectively (39). A pre-hoc power calculation showed that for a power of 0.8, alpha of 0.05, and Cohen's D of 0.8, 15 patients would be needed, and with power increased to 0.9, 19 patients would be needed. Therefore, we created a convenience sample dataset of 19 randomly selected previously unseen adult (age ≥ 18) patients for each injury type from the period between June 2019 and September 2022. The total study sample included 57 patients [42 (74%) male, 15 female], with median age of 33 [IQR: 24, 58]. Forty-seven (82%) had blunt, and 10 (18%) had penetrating injury mechanisms.

### Artificial intelligence-collaborative labeling (AICL) vs. human labeling

Human labeling strategies reflected common research practices- either (a) *de novo* annotation (i.e., no AI assistance) by a radiology attending expert with over 7 years of experience with voxelwise labeling of CT datasets, or (b) *de novo* annotation by trained research staff (a volunteer third-year medical student with one year of prior cross-sectional quantitative imaging experience), with editing of staff labels by the attending. Seed data-trained nnU-net models were used to generate labels for the 57 unseen studies, and these were also subsequently edited by the expert- a process we refer to as AI-collaborative labeling. A minimum washout period of two weeks was used between sessions, and studies were labeled in random order. De novo human labeling time and attending editing times were recorded.

Labeling was performed using 3D slicer (version 5.0.3; www.slicer.org) with the spherical threshold paint tool in the range of −20–100 Hounsfield Units to minimize noise while avoiding voxels with neighboring fat, lung, or bone. At the margins of structures with similar density, the brush size is changed or the image zoomed in as needed to ensure the label conforms to the desired contour. For hemothorax, labeling and AI inference were performed in the arterial phase as images of the chest are only acquired in this phase. For hemoperitoneum and pelvic hematoma, labeling and AI inference were performed in the portal venous phase. Labeling was conducted primarily in the axial plane with supervision and refinement in the three orthogonal planes as needed. CT volumes and segmentation masks were saved in the NIfTI and NRRD file formats respectively.

### Likert grading

A radiologist domain expert with 15 years of trauma experience was shown (1) *de novo* expert labels (2) *de novo* staff labels (3) staff labels with expert edits, (4) unedited nnU-net (“AI only”) labels, and (5) nnU-net labels with attending edits (AICL results). The radiologist was aware that, given the aim of the study, some results may involve AI and some would not but was blinded to the type and provenance of label sources and studies were shown in random order. The goal of voxelwise labeling is to achieve a trustworthy verifiable result that could be used at the clinical point of care. We define labels that meet these criteria as reaching a standard of “clinical quality”. The blinded radiologist assigned a Likert grade based on a UCLA/RAND 9-point scale, with a score of 1–3 indicating poor label quality (this was defined as requiring substantial edits for a large number of either false positive or false negative voxels, or ignoring the label and starting again from scratch), a score of 4–6 indicating moderate label quality (requiring further editing to reach clinical quality), and a score of 7–9 indicating excellent label quality requiring minimal to no edits (Table 1). The latter range is considered a clinically acceptable result. The first six consecutive patients were randomly selected from each group (hemothorax, hemoperitoneum, and pelvic hematoma) to determine whether the radiologist could distinguish between AI-only labels, and *de novo* expert labels.

**Table 1:** Segmentation label quality Likert grading system (9-point RAND/UCLA scale).

Score range	Description
Poor quality (Score 1–3)	Mostly erroneous label that does not elicit user trust. Large number of either false positive or false negative voxels. May require discounting the label and starting again given time effort required to delete false positive portions of the segmentation.
Moderate quality (Score 4–6)	Errors negatively impact interpretability/explainability and trust in volumetric results. Require further editing to reach clinically acceptable quality.
Excellent quality (Score 7–9)	Clinically acceptable label quality with explainable/high trust results. An optimal segmentation result is achievable with minimal to no editing.

### Correlations: label times, training cases, and volumes

We sought to determine to what degree *de novo* labeling times were correlated with label volumes (in milliliters) and whether attending expert editing times for staff or automated labels were decoupled from volumes. To assess internal validity of the Likert grading scale, we compare AICL to staff labels in terms of difference in Likert scales, dice similarity coefficient, and editing time- wherein higher differences in grade were expected to correlate with lower DSCs and longer edits.

### Statistical analysis

Analysis of variance (ANOVA) was used to determine a statistically significant signal within repeated measurements. Post-hoc paired signed rank tests were used to compare segmentation time and Likert grade differences. Bonferroni correction was performed to account for multiple comparisons. Correlations between label volumes and times were assessed using Pearson *r*. Spearman correlation was used for comparison involving categorical Likert grading. *p*-values < 0.05 were considered to indicate statistical significance. Statistical analysis was performed using STATA software (version 15.1).

## Results

### Results in the total sample

**Figure 2:**
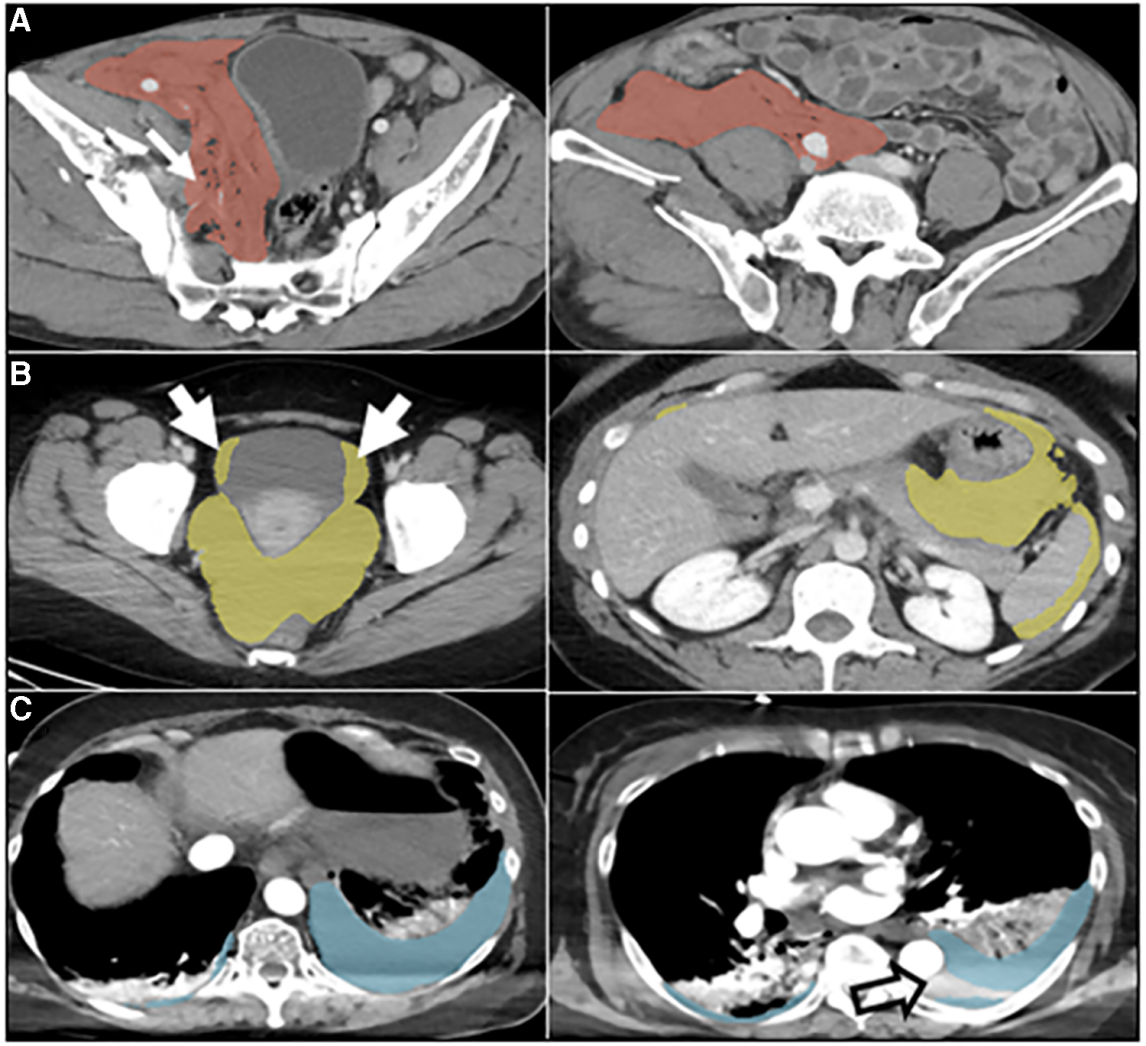
Automated labeling errors. Two slices are shown for each case example, one with an error and one without. (**A**) Pelvic hematoma (red). Arrow (left) shows extension of label into the right pelvic sidewall, requiring minimal edits. (**B**) Hemoperitoneum (yellow). Arrows show extension of label into the bladder margins, requiring minimal edits. (**C**) Hemothorax (blue). Open arrow, image right, shows unlabeled hemothorax from beam hardening artifact requiring more substantial editing. This artifact was not appreciated for hemoperitoneum or pelvic hematoma and may be related to the comparably small number of hemothorax training cases.

Median labeling times for the total sample (*n* = 57) were 788 s (s) [IQR: 525, 1,418] for *de novo* staff labels, 602 s [IQR: 368, 1,092] for *de novo* expert labels, 193 s [IQR: 113, 269] for expert edits of staff labels, and 69 s [IQR: 48, 106] for AICL (Table 2). ANOVA yielded a *p*-value < 0.0001. The AICL approach (expert edits of automated labels) reduced time effort compared to editing of staff labels by a factor of 2.8, (*p*-value < 0.0006; note: Bonferroni corrected *p*-values) (Table 3). AICL was faster than *de novo* labeling by a factor of 8.7 (expert) to 11.4 (staff) (both *p*-values < 0.0006). Examples of AI-only labeling errors addressed in AICL are shown in Figure 2. Mean Likert grades for the total sample were 8.4 (SD: 0.6) for AICL, which was significantly higher than for *de novo* expert labels [7.8 (SD 0.9), *p* < 0.0006], for edited staff labels [7.7 (SD: 0.8), *p* < 0.0006], for AI-only labels [7.8 (SD: 1.0), *p* = 0.001], and for staff labels [6.3 (SD 1.5), *p* < 0.0006]. In the 18-patient subset (6 per intracavitary hemorrhage feature, Likert score range: 5–9) presented for discrimination of AI-only and *de novo* expert labels, 12 patients were misclassified by the independent expert observer, yielding a 66% discordance rate. Correctly and incorrectly classified examples are shown in Figures 3A–C. Using the best-case scenario, AICL, as the reference standard, and worst-case scenario (staff only labels) for comparison, the difference in Likert score was inversely correlated with the DSC with r of −0.55 (*p* < 0.0001) and directly correlated to attending editing times for the staff labels with r of 0.42 (*p* = 0.001). These correlations are considered “moderate” using the Dancey and Reidy qualitative schema (40). For a Likert difference ≥4, 3, 2, 1, and 0, mean DSCs were 0.49, 0.67, 0.69, 0.65, and 0.83 respectively.

**Table 2:** Summary results for total sample and individual features.

Statistic	Vol.^a^ (ml)	Time effort (s)				Likert quality grade (1–9)				
		Expert	Staff	Staff edits	AICL	Expert	Staff	Staff edits	AI-only	AICL
Total sample (*n* = 57)										
Mean	362	752	1,092	200	90	7.8	6.3	7.7	7.8	8.4
Std dev	313	468	802	106	74	0.9	1.5	0.8	1.0	0.6
Median	274	602	788	193	69	8	6	8	8	8
IQR	[108, 494]	[368, 1,092]	[525, 1,418]	[113, 269]	[48, 106]	[7, 9]	[6, 7]	[7, 8]	[7, 9]	[8, 9]
Min	28	197	304	44	23	6	1	6	5	7
Max	1,226	2,139	4,162	512	526	9	9	9	9	9
Hemothorax (*n* = 19)										
Mean	293	561	594	147	102	8.1	7.5	7.9	7.3	8.5
Std dev	315	378	256	105	46	0.8	1.0	0.8	0.8	0.6
Median	130	422	537	101	99	8	7	8	7	9
IQR	[103, 384]	[315, 777]	[399, 725]	[75, 178]	[59, 135]	[7, 9]	[7, 8]	[7, 8]	[7, 8]	[8, 9]
Min	60	197	304	44	42	7	5	6	5	7
Max	1,226	1,749	1,429	445	186	9	9	9	8	9
Hemoperitoneum (*n* = 19)										
Mean	431	890	1,731	241	113	7.4	5.2	7.3	7.7	8.3
Std dev	328	418	932	87	109	1.0	1.6	0.6	1.1	0.5
Median	323	802	1,764	236	84	7	6	7	8	8
IQR	[152, 723]	[549, 1,346]	[929, 2,313]	[185, 270]	[49, 128]	[7, 8]	[5, 6]	[7, 8]	[7, 9]	[8, 9]
Min	39	277	537	126	29	6	1	6	6	7
Max	1,063	1,651	4,162	512	526	9	7	9	9	9
Pelvic Hematoma (*n* = 19)										
Mean	364	805	951	213	54	8.1	6.2	7.8	8.5	8.5
Std dev	279	532	566	101	20	0.8	0.9	0.9	0.6	0.6
Median	312	602	830	221	53	8	6	8	9	9
IQR	[97, 494]	[363, 1,133]	[451, 1,245]	[119, 277]	[35, 76]	[7, 9]	[6, 7]	[7, 8]	[8, 9]	[8, 9]
Min	28	259	330	57	23	7	5	6	7	7
Max	929	2,139	2,086	446	88	9	8	9	9	9

^a^ Volume is provided based on calculations from AICL.

ml, milliliters; s, seconds; Std dev, 1 standard deviation; IQR, interquartile range [Q1, Q3]; Min, minimum; Max, maximum, AICL, artificial intelligence-collaborative labeling.

“staff edits” refers to expert editing of staff labels.

**Table 3:** Corrected *p*-values for multiple comparisons- total sample and subanalyses.

Statistic	Time effort (s)				Likert quality grade (1–9)			
	Staff	Staff ed.	AICL		Staff	Staff ed.	AI-only	AICL
Total sample (*n* = 57)								
Expert	0.002	<0.0006	<0.0006	Expert	<0.0006	1.0	1.0	<0.0006
Staff		<0.0006	<0.0006	Staff		<0.0006	<0.0006	<0.0006
Staff ed.			<0.0006	Staff ed.			1.0	<0.0006
				AI-only				0.001
Hemothorax (*n* = 19)								
Expert	1	0.0006	0.0006	Expert	0.274	1.0	0.06	0.493
Staff		0.0006	0.0006	Staff		0.19	1.0	0.004
Staff ed.			0.3204	Staff ed.			0.064	0.029
				AI-only				0.002
Hemoperitoneum (*n* = 19)								
Expert	0.0066	0.0012	0.0006	Expert	0.002	1.0	1.0	0.02
Staff		0.0006	0.0006	Staff		0.001	0.002	0.001
Staff ed.			0.0114	Staff ed.			1.0	0.001
				AI-only				0.249
Pelvic hematoma (*n* = 19)								
Expert	0.8844	0.0012	0.0006	Expert	0.002	1	0.477	0.299
Staff		0.0006	0.0006	Staff		0.004	0.001	0.001
Staff ed.			0.0006	Staff ed.			0.053	0.018
				AI-only				1.0

AI-only refers to inference labels without editing.

Staff edits (abbreviated “Staff ed”) refer to expert edits of staff labels.

All *p*-values are corrected using Bonferroni method such that e.g., a *p*-value of <0.0001, becomes <0.0006.

**Figure 3A:**
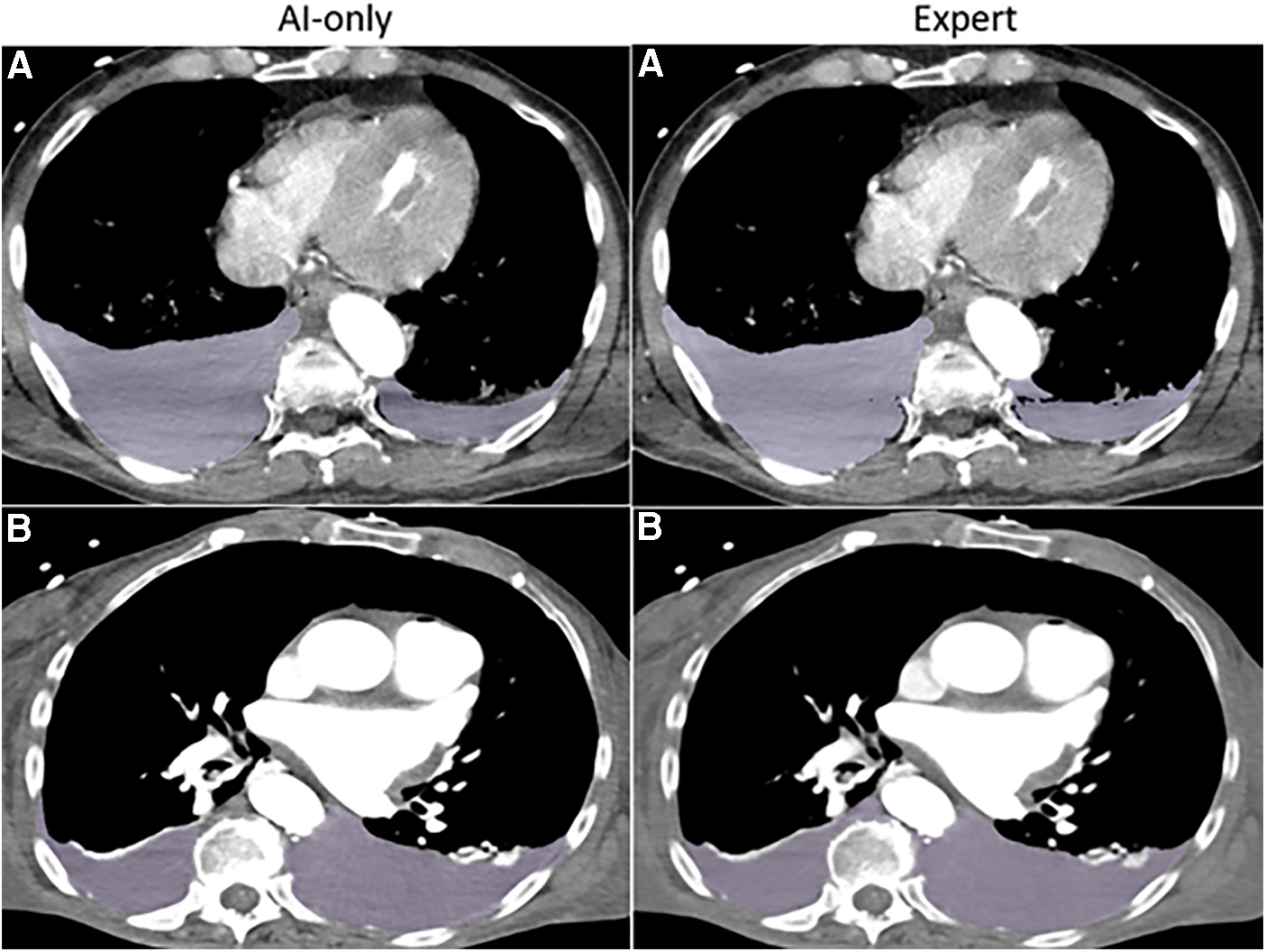
Hemothorax. The independent observer correctly classified the hemothorax label source as *AI-only* or *expert* for case **A**, but not for case **B**. Both cases received scores in the 7–9 range and were considered adequate for clinical use.

**Figure 3B:**
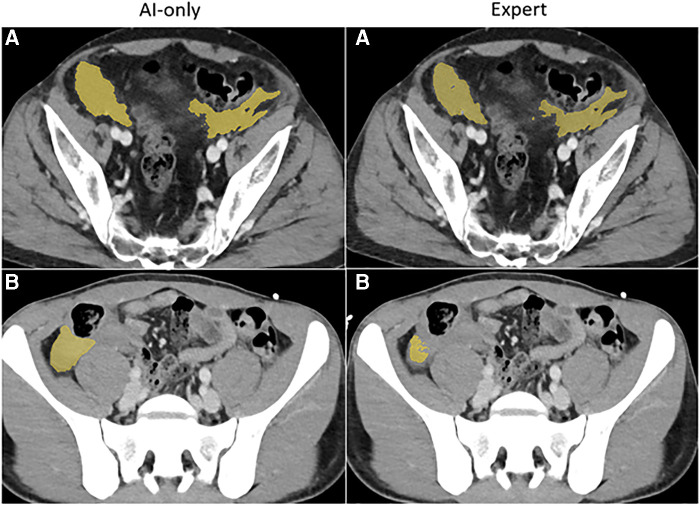
Hemoperitoneum. The independent observer correctly classified the hemothorax label sources in **A**. Both AI-only and expert labels received scores in the excellent range. In case **B**, hemoperitoneum in the right paracolic gutter is incompletely labeled. The observer believed this to be due to algorithm error.

**Figure 3C:**
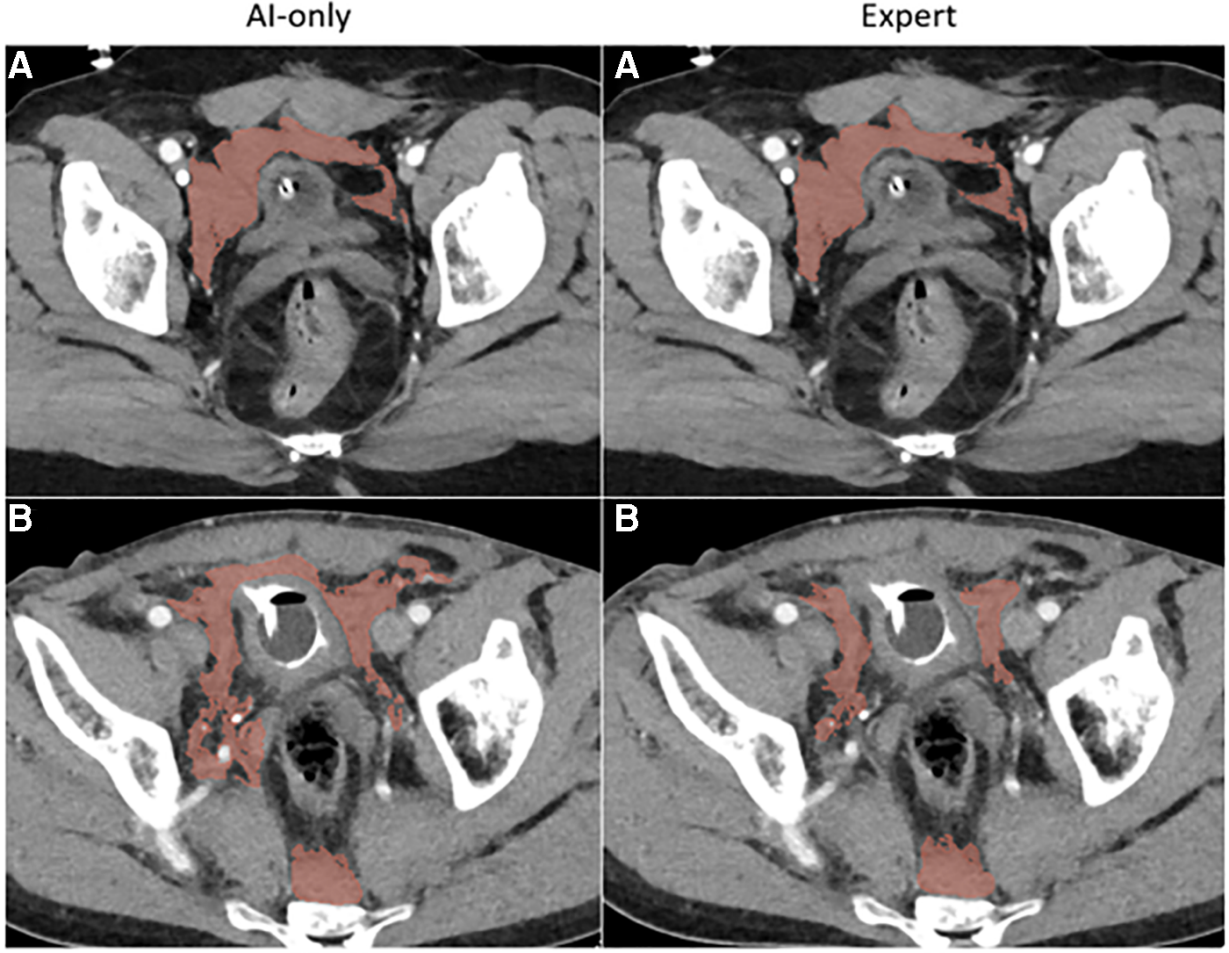
Pelvic hematoma. The independent observer correctly classified case **A**, but not case **B**. The expert annotator under-segmented blood along the right pelvic sidewall. The independent observer interpreted this as machine error.

### Hemothorax

nnU-net models were trained on manually labeled seed data with 77 patients. Among the 19 unseen hemothorax studies, AI-collaborative labels required less time effort and received the highest quality Likert grades compared with other methods. Median AICL time was 99 s [IQR: 59–135]. This was comparable to median editing time for staff labels 101 s [IQR: 75–178, *p* = 0.32], but was 4.3-fold faster than *de novo* expert labeling [422 s (IQR: 315–777), *p* = 0.0006], and 5.4-fold faster than *de novo* staff labeling [533 s (IQR: 399–725), *p* = 0.0006] (see Tables 2, 3). Likert scores for AI-collaborative labeling (mean: 8.5, SD: 0.6) were significantly higher than AI-only Likert grades (mean: 7.3, SD: 0.8, *p* = 0.002) and *de novo* staff labels (mean: 7.5, SD: 1.0, p = 0.004), and higher but comparable to *de novo* expert labels (8.1, SD: 0.8, *p* = 0.49). All AI-collaborative labels, *de novo* expert labels, and edited staff labels received Likert scores in the excellent range (scores of 7–9), whereas 3 automated labels and 2 staff labels received scores in the moderate range (scores of 4–6). Of the six studies presented for discrimination between automated and human labeling, the independent blinded observer believed that two AI-only labels were the product of human labeling, and that two of the expert labels were AI-only labels.

### Hemoperitoneum

nnU-net models were trained on labeled seed data in 130 patients. Among the 19 unseen hemoperitoneum studies, AI-collaborative labels again required the least time effort and received the highest quality Likert grades. AICL required median time effort of 84 s [IQR: 49–128]). Significantly more time effort (approximately 2.8-fold difference) was required for attending edits of staff labels [Median: 236 s (IQR: 185–270), *p* = 0.011]. Time effort for *de novo* expert [Median: 802 s (IQR: 549–1346), *p* = 0.0006], and *de novo* staff labeling [Median: 1,764 sec (IQR: 929–2,313), *p* = 0.0006] were greater than AICL by a factor of 9.5 and 21 respectively (see Tables 2, 3). Likert grades for AICL (8.3, SD: 0.5) were significantly higher than those for attending labels (mean: 7.4, SD: 1.0, *p* = 0.02), edited staff labels (mean: 7.3, SD: 0.6, *p* = 0.001), and *de novo* staff labels (mean: 5.2, SD: 1.6, *p* = 0.001). Differences between AICL and AI-only labels (mean: 7.7, SD: 1.1) did not reach significance (*p* = 0.25). All AICL labels had excellent-range Likert scores. AI-only labels, and *de novo* attending labels received scores in the moderate range in four instances each, whereas *de novo* staff labels received moderate scores in 13 studies, and poor-quality scores (range: 1–3) in three. Of the six studies presented for discrimination between automated and human labeling, the independent blinded observer again believed that two of the AI-only labels were labeled by the expert, and that two of the expert labels were AI-only labels.

### Pelvic hematoma

nnU-net models were trained on labeled seed data in 253 patients. Among the 19 patients, AICL annotations required the least time effort [Median: 53 s (IQR: 35–76)], and were 4.2-fold, 11.4-fold, and 15.7-fold faster than edited staff labels [Median: 221 s (IQR: 119–277), *p* = 0.0006], *de novo* expert labels [Median: 602 s (IQR: 363–1,133), *p* = 0.0006], and *de novo* staff labels [Median: 830 s (IQR: 451–1,245), *p* = 0.0006], respectively (see Tables 2, 3). AI-only and AICL annotations received the same mean Likert grades (Mean: 8.5, SD: 0.6). All scores for these two groups were in the excellent range (score 7–9) with discrepancy by 1 Likert grade in 4 studies, and a discrepancy by 2 grades in 1 study. Likert quality grades for AICL were significantly higher than for *de novo* staff labels (mean: 6.2, SD: 0.9, *p* = 0.001) and edited staff labels (mean: 7.8, SD: 0.9, *p* = 0.018), and were higher but similar to *de novo* expert labels (mean: 8.1, SD: 0.8, *p* = 0.30). All *de novo* expert labels were also scored within the excellent range, whereas edited staff labels were scored in the moderate range for 2 studies, and *de novo* staff labels were scored in the moderate range in 13 studies. No scores were rated in the “poor” range. Of the six studies presented for discrimination between automated and human labeling, the blinded observer believed that three AI-only labels were annotated by the expert and believed that one attending segmentation was an AI-only inference label.

### Correlations: volumes, training cases, and time effort

Volumes ranged between 59.6 and 1,225.9 ml for hemothorax; between 38.6 and 1,062.6 ml for hemoperitoneum; and between 28.1 and 929.4 ml for pelvic hematoma. In the 57 patients, Pearson correlation (*r*) for i. *de novo* expert labeling time and ii. *de novo* staff labeling time vs. target volume were *r* = 0.61 (*de novo* expert), and 0.50 (*de novo* staff). Pearson's *r* for i. AICL and ii. staff label editing time vs. target volume were r = 0.36 (AICL), and 0.59 (staff edits). *de novo* and edited staff label times were moderately correlated with segmentation volume, but AICL times and volumes were only weakly correlated (40). Large AI-inferred volumes often required short editing times when much of the pathology was already correctly “filled-in”, thus, AICL disassociates time effort from volume. This was not the case for edited staff labels. Time effort to correct staff errors (which corresponded with significantly lower mean Likert scores) increased as targets increased in size.

Although correlation does not equal causation, we observed that the tasks with more training cases had higher AI label quality and decreasing AICL time. Mean AI-only Likert grades increased by task from 7.3 to 7.5 to 8.5, and median automated editing times decreased from 99 to 84 to 53 s for hemothorax (n = 77 training cases), hemoperitoneum (*n* = 130), and pelvic hematoma (*n* = 253) respectively. Hemothorax is considered the easiest task with well-defined crescentic shape and dependent distribution but had the fewest training examples, the longest editing times, and the lowest Likert scores.

## Discussion

A paucity of labeled data is generally acknowledged as the primary bottleneck for CAD research and development (6, 41). The cost, time, and human capital needed for voxelwise annotation of hemorrhage-related or similarly complex pathology on computed tomography is tremendous (42), and this is reflected in the comparatively small datasets reported in published studies to date (4). There is a recognized need for such tools, but few have come to market (4, 32).

A variety of emerging methods, including federated learning (43), synthetic data augmentation and semi-supervised approaches (30), active learning (13), and AI-assisted annotation or contour editing (7, 16, 17) are being explored for overcoming the scarcity of labeled data in cross-sectional imaging (6). In the meantime, investigators are beginning to use collaborative human-in-the-loop strategies with supervision and editing of automated labels to accelerate voxelwise annotation (19–21). In a recent COVID CT infiltrate segmentation challenge, automated labels edited by experts were used as the reference standard (19). In a pilot study with a maxillary sinus segmentation use case, nnU-net inference labels were edited and used for iterative retraining in several rounds, with final results compared to expert labels as the ground truth (21). Preliminary data from our early experience with deep learning-based pelvic hematoma segmentation showed that our model ignored outlier labeling errors, suggesting that modest overlap metrics for this complex feature could be due in part to an imperfect reference standard. AICL was proposed as a means of overcoming both the time effort and fallibility associated with manual labeling for difficult tasks requiring high cognitive load. Such a strategy could potentially lead to higher agreement but lower quality due to automation bias. To our knowledge, this had yet to be explored using semi-quantitative grading of label quality.

Labeling of Intracavitary pooled hemorrhage such as hemothorax, hemoperitoneum, and pelvic hematoma poses similar challenges to COVID infiltrates and surveillance of advanced malignancy; volumes can vary greatly, sometimes exceeding a liter of fluid for a given body cavity. Other challenges include the often multifocal, irregular, and ill-defined characteristics of pooled unopacified blood, and small differences in contrast with neighboring structures, including viscera and body wall (25, 29, 34).

CT is the routine imaging workhorse for patients with major trauma (44–46). Exsanguination remains a leading cause of preventable death, but clinical indices such as the shock index (SI) or Assessment of Blood Consumption (ABC) score are only modestly predictive and insensitive for life-threatening blood loss (47–49). The ability to predict outcomes or personalize treatment based on granular measurements of hemorrhage-related pathology on CT is a major precision medicine and personalized treatment-related value proposition of automated segmentation tools in the trauma domain (25, 30, 31, 34–36, 50–53), but large scale out-of-sample studies are required to confirm proof-of-principle.

In this work, we found that AICL for hemothorax, hemoperitoneum, and pelvic hematoma resulted in an overall 2.8-fold reduction in time effort over editing of staff labels, and an 8.7-fold reduction in time effort compared to *de novo* manual labeling by an expert. Further, AICL had significantly higher quality Likert scores (mean 8.4) than human *de novo* or edited labels (means from 6.3 to 7.8, *p* = 0.02 to < 0.001). For staff labels, increasing deviations in Likert grades from AICL grade as the reference standard correlated with lower DSC and longer editing times. The domain expert performing Likert grading was unable to correctly discriminate between manual expert labels and AI-only labels in the majority of patients, with an overall discordance rate of 66%.

Based on these results, the use of AICL appears justified as a reference standard for future experiments with our models and expert annotator. Such an approach can be extrapolated to very large datasets to substantially reduce costs and human capital as model-derived labels can be edited in place of those from trained staff. Our results do not necessarily generalize to other use cases or research teams and are not meant to justify using AICL as ground truth without appropriate quality control. However, our quality assessment framework could be used by other investigators and for other use cases to evaluate AICL annotation as an optimal high-throughput, high-quality reference standard. This would facilitate scaling voxelwise annotation to large cross-sectional imaging datasets for large-scale experiments. Once high-quality data annotation is complete and algorithms trained, they can be shadow-tested in the clinical environment with PACS-integrated software (54).

Before using AICL labels as ground truth, three conditions should ideally be met:

1. *AICL should take less time effort than human labeling or editing of human labels*. To achieve this, we recommend that sufficient manually-labeled seed data are used to generate at least moderate quality AI-only labels, using the proposed grading scale. All grading should be performed by a blinded observer with relevant clinical domain expertise not involved in the labeling process. The number of manually labeled studies used as seed data will vary by task and will need to be determined on an individual basis.
2. *Semi-quantitative quality scores for AICL should be improved compared with human labels*. This ensures that automation or complacency bias does not adversely affect label quality.
3. *The external blinded observer performing Likert grading should have difficulty correctly discriminating AI and human labels*. This serves as an additional indicator that the AI-only labels are of sufficient quality to result in substantial time savings when using AICL. The proposed Likert grading system can also be employed as a preliminary assessment of the readiness of segmentation models for “in-the-wild” deployment.

Our study has several limitations. We were primarily constrained by the effort required from multiple participants in four different scenarios. Consequently, a small number of unseen studies were included. Models were trained using historical data from the same institution. Further, different numbers of patients were used to train each model. The pelvic hematoma model (trained with 253 CTs, compared to 130 for hemoperitoneum, and 77 for hemothorax), had the shortest editing times and highest Likert scores. Although we are unable to establish with certainty the degree to which this is related to more training cases, considering that pelvic hematoma is highly irregular and multi-compartmental, it is our experience that this represents a substantially more challenging task than labeling of hemothorax (compare for example Figures 3A,C). Based on comparative task simplicity alone, we would expect the hemothorax nnU-net model to yield the highest rather than the lowest quality labels. Additionally, our study was only powered to detect differences in time effort. However, differences in Likert scores were still found to be significant.

In the future, our approach could likely be combined with active learning and semi-automated AI-assisted annotation for further improvements in throughput.

## Conclusion

The precision-medicine-related value of volumetric imaging has been explored in a variety of domains such as surveillance for cancer progression, COVID infiltrate quantification, and hemorrhage burden in trauma. However, studies typically have insufficient sample sizes to ensure model generalizability and robustness in the clinical setting, which limits opportunities for productization and regulatory approval. The time effort and human capital required for manual voxelwise labeling prevents scaling to large datasets, particularly for pathology with irregular, multifocal, and potentially very large targets. Further, manual labels suffer from human error and are an imperfect ground truth.

We evaluated an AICL approach using models initialized on manually-labeled seed data. AICL annotations met three conditions for use as a reference standard: 1. They required significantly less time effort than human labeling, with time savings approaching an order of magnitude. 2. Semi-quantitative quality scores were improved compared with human-only labels, and 3. Automated and manual labels were not readily distinguishable. When these conditions are met, edited automated labels can be considered a high-quality gold standard for rapid curation of voxelwise data. Further improvements in throughput and performance may be possible with human-in-the-loop iterative retraining, interactive semi-automated labeling (AI-assisted annotation), and active learning strategies.

## Data Availability

The raw data supporting the conclusions of this article will be made available by the authors, without undue reservation.
